# The Role of Tranexamic Acid in Sleeve Gastrectomy: A Systematic Review and Meta-Analysis

**DOI:** 10.7759/cureus.54269

**Published:** 2024-02-15

**Authors:** Abdulkreem Al-Juhani, Galal F Sharaf, Saeed Aseri, Hadeel Alosaimi, Shaden A Alharkan, Joud K AlGhamdi, Faris S Hariri, Lojain Daak, Ikhlas Daak

**Affiliations:** 1 General Surgery, King Abdulaziz University Faculty of Medicine, Jeddah, SAU; 2 General Surgery, University of Queensland, Cairo, EGY; 3 Psychiatry, King Abdulaziz University Faculty of Medicine, Jeddah, SAU; 4 Surgery, University of Tabuk, Tabuk, SAU; 5 Medicine and Surgery, Arabian Gulf University, Manama, BHR; 6 Medicine, King Abdulaziz University Hospital, Jeddah, SAU; 7 Medicine, Batterjee Medical College, King Abdulaziz University Hospital, Jeddah, SAU; 8 Medicine and Surgery, Jazan University, Jazan, SAU; 9 Medicine, Jazan College of Medicine and Medical Sciences, Jazan University, Jazan, SAU

**Keywords:** compression staple, unprovoked venous thromboembolism, #laparoscopic sleeve gastrectomy, laparoscopic sleeve gastrectomy complications, intravenous tranexamic acid

## Abstract

Tranexamic acid (TXA) is an essential procoagulant drug used in various intra- and postoperative situations. Its efficacy and safety profile in obese cases undergoing laparoscopic sleeve gastrectomy (LSG) is still unresolved. Therefore, this meta-analysis evaluated and investigated the current intra- and postoperative effects and hazards of TXA on patients undergoing LSG. As for methodology, Web of Science, Cochrane Library, Scopus, and PubMed were thoroughly searched for relevant studies. Retrieved results were prepared for screening through Endnote, helping to identify eligible studies. Relevant patient characteristics and outcomes were extracted. The methodological quality of the relevant studies was appraised using the respected appraisal tool. Six studies of different designs were enrolled, comprising 753 cases that underwent LSG and administered TXA. Their mean BMI and age went from 37.3 to 56.25 kg/m^2 ^and 33.5 to 43.25 years, respectively. Tranexamic acid significantly linked to reduction in intraoperative bleeding instances, operative blood loss, and operative duration, compared to placebo ((RR = 0.66, 95% CI [0.44, 0.98], P=0.04, I^2^ = 81%); (MD = -39.64, 95%CI [-75.49, -3.78], P=0.03, I^2^=94%); (MD=-5.84, 95%CI [-9.62, -2.05], P=0.003, I^2^=73%)). Tranexamic acid also significantly showed superiority regarding postoperative bleeding events and duration of hospitalization compared to the control group ((RR= 0.45, 95%CI [0.29, 0.69], P=0.0002, I^2 ^=0%); (MD=-0.24, 95%CI [-0.32, -0.17], P< 0.0000, I^2 ^=0%)). Moreover, follow-up of the enrolled patients for a minimum of three to six months resulted in no reported thromboembolic instances, suggesting a negligible risk for thromboembolism among patients undergoing LSG and receiving TXA. In conclusion, tranexamic acid demonstrates a robust safety and efficacy profile for its use in patients undergoing LSG, with no reported instances of thromboembolism. Variations in TXA administration regimens, bleeding definitions, procedural techniques, and potential confounding medications could not be accounted for, necessitating additional large-scale RCTs to address and bridge knowledge gaps.

## Introduction and background

The escalating prevalence of obesity corresponds with an increment in metabolic surgeries, necessitating a rise in perioperative complications, including bleeding [1]. Managing bleeding and preventing venous thromboembolic (VTE) instances are significant challenges in metabolic surgery [2,3]. Around 3% of obese cases subjected to laparoscopic sleeve gastrectomy (LSG) experience bleeding occurrences after surgery [4-9]. Early mobilization of patients, a characteristic of fast-track metabolic surgery, was proven effective and safe for decreasing VTE instances [10,11]. However, the early discharge of patients also limits the clinical window for detecting hemorrhages, demanding reduction of bleeding after fast-tracking metabolic surgeries. 

Various parameters for preventing hemorrhage have been explored, including staple height, the timing of stapler closure before stomach dissection, and different staple line reinforcement (SLR) procedures [12]. Despite the various elements studied, none significantly reduced the bleeding probability [7,13,14]. Tranexamic acid (TXA) has effectively reduced bleeding in surgical fields, such as orthopedic and cardiothoracic surgery, without elevating the likelihood of VTE occurrences [15-17]. Klaassen and the associated team scrutinized the postoperative benefits of TXA regarding hemorrhagic complications in 44 patients and found no reoperations were required in 40 patients, and no VTE instances were observed [8]. In a non-randomized study, 25 patients who underwent LSG and received TXA had fewer bleeding sites demanding lower hemostatic clips, lower volume of bleeds, and less surgery time than a control group of 25 patients. Given the affordability of TXA, the researchers concluded that its administration was a simple and cost-effective solution [18]. 

Lately, Mocanu and his team carried out a systematic review and meta-analysis that included 571 obese patients who had undergone LSG. They discovered a significant correlation between TXA and reduced postoperative bleeding compared to the control group [19]. However, the studies they enrolled in were of moderate to low quality, including one randomized controlled trial (RCT) with a high risk of bias [20]. Recently, Sermet and Hart conducted a high-quality RCT involving a total of 278 obese patients who had undergone LSG. They compared TXA with a placebo regarding safety and efficacy outcomes, including intraoperative bleeding, postoperative bleeding, and thromboembolic events [21,22]. Consequently, we carried out this systematic review and meta-analysis to thoroughly evaluate the latest available evidence regarding the safety and efficacy outcomes of TXA when administered to obese patients undergoing LSG.

## Review

Methods

This systematic review and meta-analysis (SRMA) was executed with guidance from the latest Cochrane Handbook [23] and was reported tracking the instructions of the latest Preferred Reporting Items for Systematic Reviews and Meta-Analyses (PRISMA) [24].

Search Strategy

A comprehensive search query was developed to search Scopus, Cochrane Library, PubMed, and Web of Science, since their establishment until December 2023 using the subsequent terms: (("Tranexamic acid" OR "cyklokapron" OR amcha OR "trans-4-Aminomethyl -cyclohexanecarboxylic Acid" OR "t-AMCHA" OR amca OR anvitoff OR cyklokapron OR ugurol OR kabi 2161 OR spotof OR transamin OR amchafibrin OR exacyl OR tranexamsaeure OR tranexa OR lysteda) AND (Gastrectom*)). Previous terms were edited to fit the syntax of each searched database. Results were compiled in the EndNote software (Clarivate, London, UK) to remove duplicates, and the remaining records were exported to an Excel sheet (Microsoft Corporation, Redmond, USA) for screening.

Inclusion and Exclusion Criteria

The retrieved English articles were eligible if they matched these criteria: Population: adults and elderly with Body mass index (BMI) ≥ 30 who underwent laparoscopic sleeve gastrectomy (LSG); Intervention: tranexamic acid; Control: placebo; Outcomes: intraoperative bleeding, operative duration, postoperative blood loss, hospital stay, and pre-postoperative hemoglobin change. Non-English, reviews, and animal studies were excluded.

Data Extraction

The eligible studies were summarized through the following data: Study ID (first author's last name/publication year, study arms n,(%), site, study design, age in years,(mean ± SD), body mass index (BMI)(mean ± SD), males n,(%), follow up duration (months), comorbidities n,(%), haemoglobin (HB)(g/dl) (mean ± SD), procedure and techniques, TXA dose and administration route, time of administration, inclusion criteria, primary endpoints, and conclusion. Additionally, these outcomes were extracted: operative time, intraoperative bleeding events, the volume of intraoperative blood loss (ml), number of used clips of hemostasis, postoperative bleeding events, change in pre-postoperative hemoglobin, length of hospital stay, and venous thromboembolic (VTE) occurrences.

Risk-of-Bias Assessment

The methodological quality of the included studies was appraised by either the first version of the Cochrane risk of bias (ROB) tool [25], the methodological index for non-randomized studies (MINORS) [26], or the observational studies assessment tool developed by the National Institutes of Health (NIH) [27]. The Cochrane ROB tool consists of seven domains that assess selection, performance, detection, attrition, and selective reporting biases. Each domain is judged by either a high, low, or unclear risk of bias. The MINORS appraisal tool consists of seven domains, including clarified aim, study population, prospective gathering of the data, appropriate follow-up period, and extent of lost data during follow-up. Each of the seven domains is given a score of either 0, 0.5, or 1 point. Appraised studies are considered of overall poor, fair, or good quality if obtain an overall score of (0 - 3.5 points), (4 - 5.5 points), or (5.5 - 7 points), respectively. The NIH assessment tool judges observational studies through 14 questions assessing various domains including clarified objective, unified study population, sample size justification, exposure, outcomes, follow-up, and statistical analysis. Each question is given a score of either 0, 0.5, or 1 point; each study is judged as either poor (0-7 points), fair (7.5-10.5 points), or high quality (11-14 points) according to the overall assessment points.

Data Analysis

Meta-analysis was conducted using Review Manager (RevMan), version 5.4 (Cochrane, London, UK). We used Mean and 95% confidence interval to present continuous data and risk ratio and 95% confidence interval to present the categorical data. Heterogeneity among the pooled studies was quantified using the I-squared (I2) test, and any heterogeneity was determined using the P-value of the Chi-square test. Heterogeneity was considered significant if the I-squared and the P-value of Chi-squared tests valued > 50% or < 0.1, respectively. We used the random effect model for heterogeneous data, while the fixed effect model was applied if the data was homogeneous.

Results

Literature Search and Study Selection

**Figure 1:**
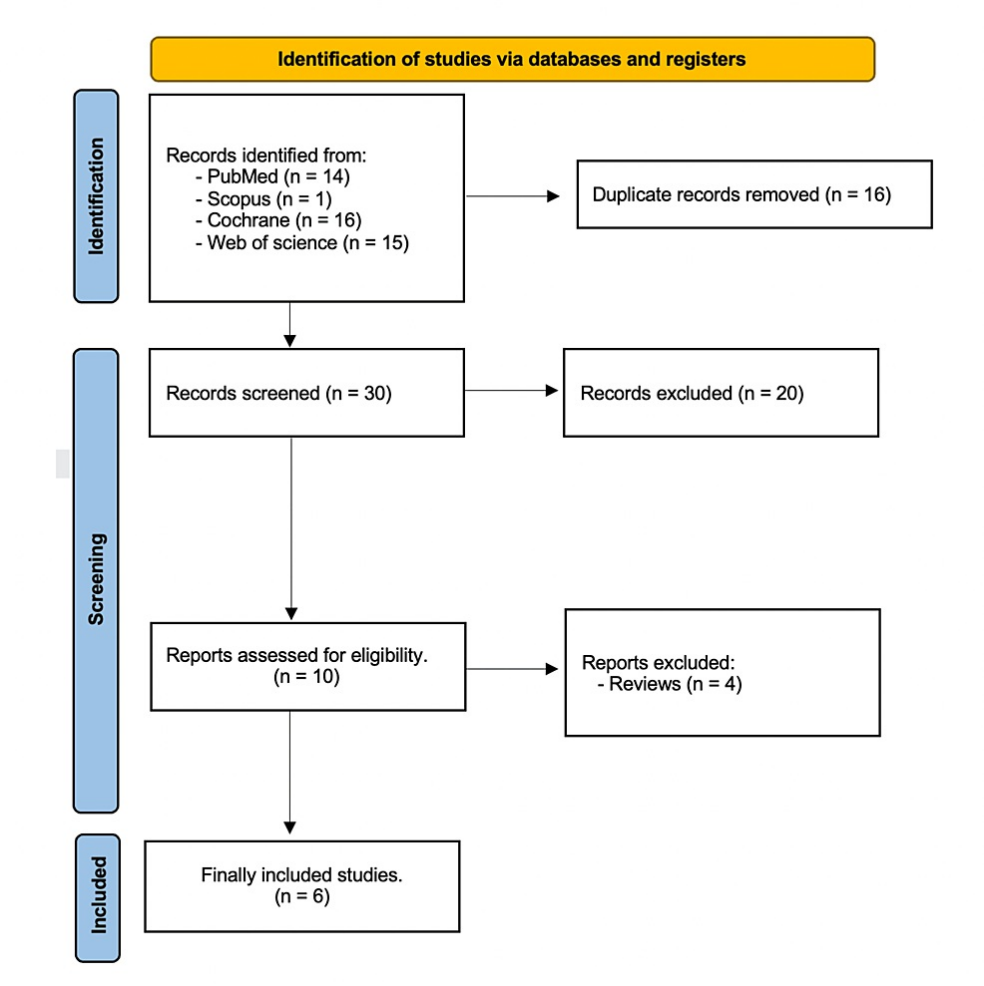
PRISMA flow chart PRISMA: Preferred Reporting Items for Systematic Reviews and Meta-Analyses

Initial searching through Scopus, Cochrane Library, PubMed, and Web of Science resulted in 46 studies, of which 16 duplicates were found and omitted. Thirty studies were screened by titles and abstracts, 10 studies were eligible for full-text screening, and six studies were ultimately eligible for our review [18,20-22,28,29] (Figure 1).

Study Characteristics of the Included Studies

Our SRMA compiled data from six studies conducted in Kuwait, Brazil, the United Kingdom, Poland, the Netherlands, and Türkiye. We compiled a total sample of 753 and followed up for a minimum of three to six months. Their mean BMI spanned between 37.3 and 56.25 kg/m2, their reported mean age ranged from 33.5 to 43.25 years, and females represented around 80% of the whole enrolled population. More detailed patient characteristics are exhibited in Table 1.

**Table 1:** Patient characteristics Abbreviations: RCT= Randomized clinical trial; UK= United Kingdom; DM= Diabetes Mellitus; HTN= Hypertension; SJP= Severe joint pain; BMI= Body Mass Index; LSG= Laparoscopic Sleeve Gastrectomy; TXA= Tranexamic acid; OSAS= obstructive sleep apnea syndrome; OSA= obstructive sleep apnea

Study ID	Study arms, n(%)	Site	Study design	Age, (mean±SD)y	BMI(kg/m2), (mean±SD)	Male, n(%)	Follow up duration (months)	Comorbidities, n(%)	Hemoglobin, (mean±SD) (g/dl)	Procedure and techniques	TXA dose and route	Administration time	Inclusion criteria	Primary endpoints	Conclusion
Alhomoud et.al 2016 [20]	Tranexamic acid, 25(50)	Kuwait	RCT	-	-	8(16)	Four	-	-	-	10 mg/kg, intravenous	Induction	1. From September 2014 to December 2014 2. Underwent laparoscopic sleeve gastrectomy	1. Intra-operative blood loss 2. Post-operative blood loss	"Tranexamic acid is an antifibrinolytic agent that inhibits the action of plasmin. There is also reduction in blood level of D-dimer. It is seen to significantly reduce intraoperative blood loss during surgery. Additionally, there seems to be no alterations of coagulation parameters or untoward systemic effects. This should prompt further trials"
	Control, 25(50)														
Brito et.al 2022 [29]	Tranexamic acid, 30(49.18)	Brazil	Non-RCT	37.67 ± 2.17	38.1 ± 1.075	11(36.7)	At least Six	-	13.1 ± 0.3	LSG 32 Fr Bougie Echelon Flex Endopath (green, gold, blue, non-buttressed)	1 g intravenous	Induction	1. Patients aged 18 to 65 years old 2. Physical status score of II or III 3. Undergoing bariatric surgery and LSG 4. From January 2019 to June 2020	1. Hemodynamic variables 2. Hematological variables	"The use of tranexamic acid was effective in reducing bleeding rates and of hospital stay length, in addition to demonstrating the clinical safety of its use, for not having been associated with any thromboembolic events"
	Control, 31(50.82)			39.55 ± 1.71	37.3 ± 0.7	5(16.1)			12.3 ± 0.2						
Chakravartty et.al 2016 [18]	Tranexamic acid, 25(50)	UK	Prospective cohort study	43.25 ± 13.15	55 ± 11	5(20)	-	1. DM, 5(20)	-	LSG 38 Fr Bougie Echelon Flex Endopath (green, gold, blue, non-buttressed)	1 g intravenous	Induction	1. Patients undergoing sleeve gastrectomy 2. Operated on by one of two experienced surgeons, with identical anesthetic regiments, operative steps, and postoperative protocols	1. Total bleeding points 2. Overall morbidity 3. Overall mortality	"Intraoperative prophylactic tranexemic acid use is a simple and economical option for effectively reducing staple line bleeds leading to significant decrease in operating times"
								2. HTN, 8(32)							
								3. Hyperlipidemia, 5(20)							
								4. Sleep apnea, 1(4)							
								5. Others, 2(8)							
	Control, 25(50)			36 ± 9	56.25 ± 8.25	5(20)		1. DM, 2(8)							
								2. HTN, 4(16)							
								3. Hyperlipidemia, 4(16)							
								4. Sleep apnea, 2(8)							
								5. Others, 3(12)							
Lech et.al 2022 [28]	Tranexamic acid, 157(50)	Poland	Retrospective cohort study	41.6 ± 11.5	46 ± 9.6	33(21)	At least Six	1. DM, 33(21)	-	LSG 36 Fr Bougie Endo GIA (3 purple, 2 blue cartridges, non-buttressed)	1 g intravenous	Induction and post-operatively every 8 h for 3 doses	1. Patients undergoing LSG 2. 1 g of intravenous TXA was routinely administered as an induction to all LSG patients and three times after the surgery every 8 h 3. They gave written informed consent	1. Bleeding events 2. Change in Hemoglobin	"The prophylactic doses of TXA may be useful in reducing the hemorrhagic events during LSG. It may also shorten the length of hospital stay and the operating time"
								2. HTN, 73(46.5)							
	Control, 157(50)			39 ± 11.2	44.6 ± 6.6	33(21)		1. DM, 26(16.6)	-						
								2. HTN, 67(42.7)							
Hart et.al 2023 [22]	Tranexamic acid, 49(48.51)	Netherlands	RCT	36 ± 10.9	42.3 ± 5.4	11(22.4)	At least Three	1. HTN, 8(16.3)	14.18 ± 1.29	LSG; hemostatic clips for active bleeding at the staple line. Fibrin sealant use peroperative Staple device Cartridge GOLD (3.0 mm) Cartridge BLUE (2.4 mm)	1.5 g intravenous	Induction	1. All patients Planned to undergo primary SG 2. With sufficient Dutch or English language proficiency 3. Gave written informed consent	1. Bleeding events 2. Hemostasis clips during surgery	"This study did not demonstrate a statistically significant difference in use of hemostatic clip devices and major complications after preoperative administration of TXA. However, TXA seems to have positive effects on clinical parameters, minor complications, and LOS in patients undergoing SG, without increasing the risk of VTE. Larger studies are needed to investigate the effect of TXA on postoperative major complications"
								2. DM, 2(4.1)							
								3. Dyslipidemia, 5(10.2)							
								4. OSA, 17(34.7)							
								5. SJP, 8(16.3)							
								6. GERD, 4(8.2)							
	Control, 52(51.49)			36.8 ± 12.3	41.5 ± 5.3	10(19.2)		1. HTN, 12(23.1)	14.02 ± 0.97						
								2. DM, 3(5.8)							
								3. Dyslipidemia, 5(9.6)							
								4. OSA, 18(34.6)							
								5. SJP, 8(15.4)							
								6. GERD, 5(9.6)							
Sermet et.al 2023 [21]	Intra-operative Tranexamic acid, 60(33.9)	Turkey	RCT	35.8 ± 10.25	41.4 ± 2.05	14(23.33)	At least Six	1. OSAS, 6(10)	13.2 ± 3.2	A 38-French gastric bougie (reservoir tube used for stomach calibration) was placed for gastric calibration	1 g intravenous	Induction and post-operatively	1. Patients aged 18–65 years 2. BMI> 40–49.9 kg/cm2 3. No anticoagulant use, and no previous bariatric surgery	1. Intra-operative blood loss 2. Post-operative blood loss	"This study provides evidence that TXA administered during LSG is effective in reducing postoperative bleeding. No data were obtained regarding the superiority of TXA administration at the beginning of induction and at the end of surgery"
								2. DM, 6(10)							
								3. HTN, 11(18.3)							
								4. Other diseases, 8(13.3)							
	Post-operative Tranexamic acid, 59(33.33)			34.6 ± 9.25	42.1 ± 1.93	11(18.64)		1. OSAS, 4(6.7)	13.4 ± 3.1						
								2. DM, 10(16.9)							
								3. HTN, 7(11.9)							
								4. Other diseases, 9(15.2)							
	Control, 58(32.77)			33.5 ± 10.5	42.6 ± 1.925	13(22.41)		1. OSAS, 5(8.6)	13.2 ± 3.1						
								2. DM, 9(15.5)							
								3. HTN, 8(13.8)							
								4. Other diseases, 8(13.8)							

Methodological Quality Assessment

The design of the included studies was either RCT, cohort, or non-randomized trial. Appraisal of the cohort studies yielded an overall fair quality [18,28]. Brito and the associated team [29] conducted a non-randomized trial of overall good quality; the only domain with not-reported data was the inclusion of consecutive patients' domain (Tables 2, 3).

**Table 2:** National Institutes of Health (NIH) quality assessment tool for observational cohort and cross-sectional studies

ID	NIH Quality Assessment Tool for Observational Cohort and Cross-Sectional Studies																Quality rating: Good (11-14) or fair (7.5-10.5) or poor (0-7), Yes = 1 // No = 0.5 // NR & NA & CD = 0
	1. Was the research question or objective in this paper clearly stated?	2. Were eligibility/selection criteria for the study population prespecified and clearly described?	3. Were the participants in the study representative of those who would be eligible for the test/service/intervention in the general or clinical population of interest?	4. Were all eligible participants that met the prespecified entry criteria enrolled?	5. Was the sample size sufficiently large to provide confidence in the findings?	6. For the analyses in this paper, were the exposure(s) of interest measured prior to the outcome(s) being measured?	7. Was the time frame sufficient so that one could reasonably expect to see an association between exposure and outcome if it existed?	8. For exposures that can vary in amount or level, did the study examine different levels of the exposure as related to the outcome (eg, categories of exposure, or exposure measured as continuous variable)?	9. Were the exposure measures (independent variables) clearly defined,valid, reliable, and implemented consistently across all study participants?	10. Was the exposure(s) assessed more than once over time?	11. Were the outcome measures prespecified, clearly defined, valid, reliable, and assessed consistently across all study participants?	12. Were the people assessing the outcomes blinded to the participants' exposures/interventions?	13. Was the loss to follow-up after baseline 20% or less? Were those lost to follow-up accounted for in the analysis?	14. Were key potential confounding variables measured and adjusted statistically for their impact on the relationship between exposure(s) and outcome(s)?	Total scores		
	Yes / No / Not reported (NR) or cannot determine (CD) or not applicable (NA)	Yes / No / Not reported (NR) or cannot determine (CD) or not applicable (NA)	Yes / No / Not reported (NR) or cannot determine (CD) or not applicable (NA)	Yes / No / Not reported (NR) or cannot determine (CD) or not applicable (NA)	Yes / No / Not reported (NR) or cannot determine (CD) or not applicable (NA)	Yes / No / Not reported (NR) or cannot determine (CD) or not applicable (NA)	Yes / No / Not reported (NR) or cannot determine (CD) or not applicable (NA)	Yes / No / Not reported (NR) or cannot determine (CD) or not applicable (NA)	Yes / No / Not reported (NR) or cannot determine (CD) or not applicable (NA)	Yes / No / Not reported (NR) or cannot determine (CD) or not applicable (NA)	Yes / No / Not reported (NR) or cannot determine (CD) or not applicable (NA)	Yes / No / Not reported (NR) or cannot determine (CD) or not applicable (NA)	Yes / No / Not reported (NR) or cannot determine (CD) or not applicable (NA)	Yes / No / Not reported (NR) or cannot determine (CD) or not applicable (NA)			
Chakravartty et.al 2016 [18]	Yes	Yes	Yes	NR	NR	Yes	Yes	NR	Yes	NR	NR	No	Yes	Yes	8.5	Fair	
Lech et.al 2022 [28]	Yes	Yes	Yes	NR	NR	Yes	Yes	NR	Yes	NR	Yes	No	Yes	Yes	9.5	Fair	

**Table 3:** MINORS criteria for assessment of non-randomized clinical trial MINORS: methodological index for non-randomized studies

ID	MINORS Criteria for assessment of Non-Randomised clinical trial								
	1. A stated aim of the study?	2. Inclusion of consecutive patients?	3. Prospective collection of data?	4. Endpoint appropriate to the study aim?	5. Unbiased evaluation of endpoints?	6. Follow-up period appropriate to the major endpoint?	7. Loss to follow-up not exceeding 5%?	Total scores: Yes = 1 // No = 0.5 // NR & NA & CD = 0	Quality rating: Good (5.5-7) or Fair (4-5.5) or Poor (3.5-0)
	Yes / No / Not reported (NR) or cannot determine (CD) or not applicable (NA)	Yes / No / Not reported (NR) or cannot determine (CD) or not applicable (NA)	Yes / No / Not reported (NR) or cannot determine (CD) or not applicable (NA)	Yes / No / Not reported (NR) or cannot determine (CD) or not applicable (NA)	Yes / No / Not reported (NR) or cannot determine (CD) or not applicable (NA)	Yes / No / Not reported (NR) or cannot determine (CD) or not applicable (NA)	Yes / No / Not reported (NR) or cannot determine (CD) or not applicable (NA)		
Brito et.al 2022 [29]	Yes	NR	Yes	Yes	Yes	Yes	Yes	6	Good

**Figure 2:**
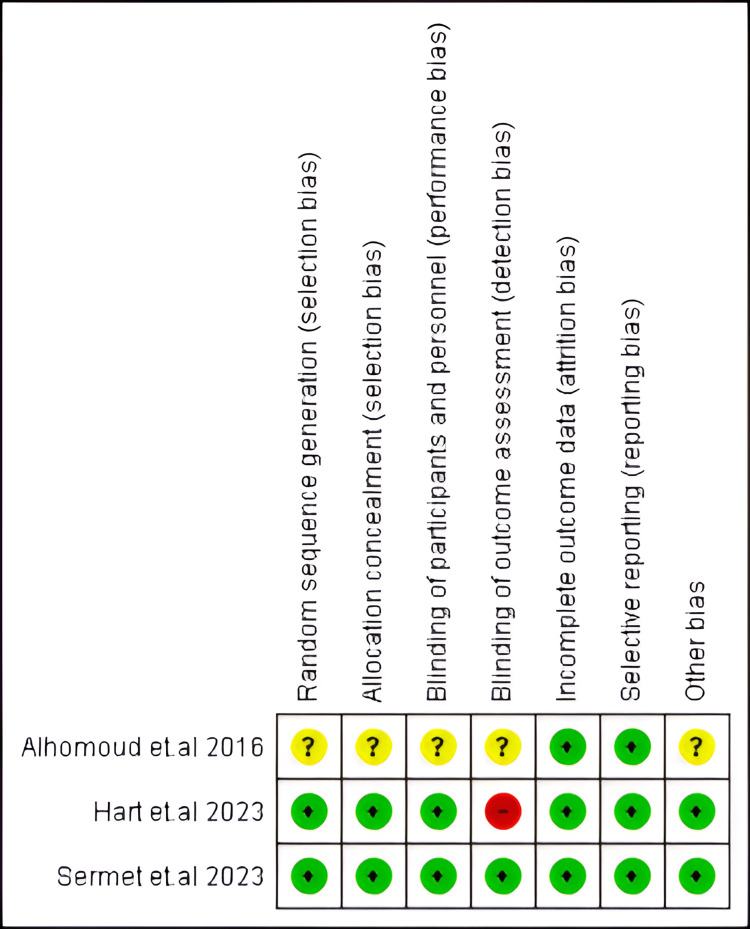
Risk of bias for our included RCTs Included studies [20-22]

Regarding the appraised RCT, Sermet and Ozsoy achieved a low risk of bias regarding all assessed domains [21]. Likewise, Hart et al., except in the detection bias domain, was of high risk [22]. Alhomoud [20] achieved low-risk attrition and reporting biases but unclear risk in all other domains (Figure 2).

Quantitative analysis

Intraoperative Outcomes

**Figure 3:**
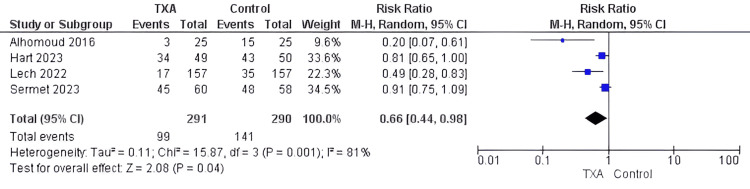
Forest plot of incidence of bleeding events (intraoperative) Included studies [20-22, 28] TXA= tranexamic acid

Bleeding events: The pooled data of 581 patients reported in four studies [20-22,28] showed that the bleeding events were significantly reduced in the group administered TXA compared to the control group (RR=0.66, 95% CI [0.44, 0.98], P=0.04). Substantial heterogeneity was found among the compiled studies but could not be resolved (I2 = 81%) (Figure 3).

**Figure 4:**
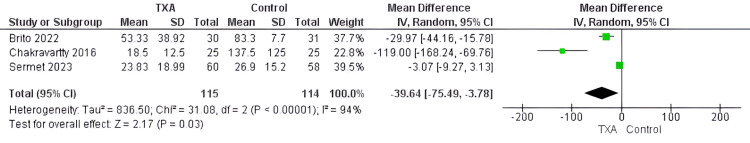
Forest plot of blood loss (ml) (intraoperative) Included studies [18, 21, 29] TXA= tranexamic acid

Blood loss (ml): Three studies with a total of 229 patients [18,21,29] showed that the group administered TXA had a significantly lower volume of lost blood intraoperatively compared to the control group (MD= -39.64, 95%CI [-75.49, -3.78], P=0.03). Considerable heterogeneity was found and could not be resolved (I2=94%) (Figure 4).

**Figure 5:**
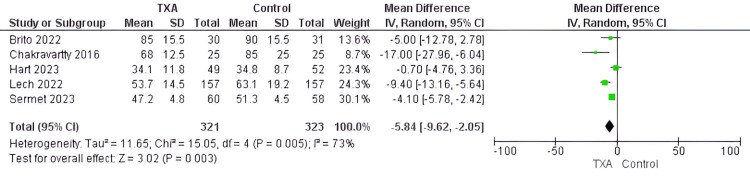
Forest plot of operative time (minutes) Included studies [18,21,22,28,29] TXA= tranexamic acid

Operative time (minutes): Five studies with substantial heterogeneity provided data from 644 patients [18,21,22,28,29] that revealed significantly shorter operative time in the TXA group compared to the control group (MD= -5.84, 95%CI [-9.62, -2.05], P=0.003, I2=73%) (Figure 5).

**Figure 6:**
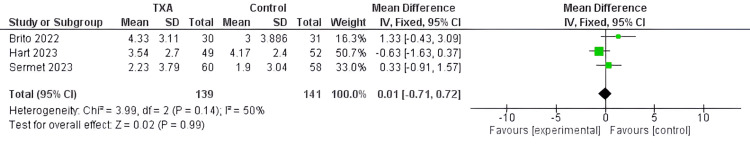
Forest plot of the number of clips of hemostasis Included studies [21,22,29] TXA= tranexamic acid

Number of clips of hemostasis: We found no significant difference between the TXA and the control groups regarding the number of clips of hemostasis according to pooling 280 patients' data from three [21,22,29] moderately heterogeneous studies (MD=0.01, 95%CI[-0.71, 0.72], P=0.99, I2=50%) (Figure 6).

Postoperative Outcomes

**Figure 7:**
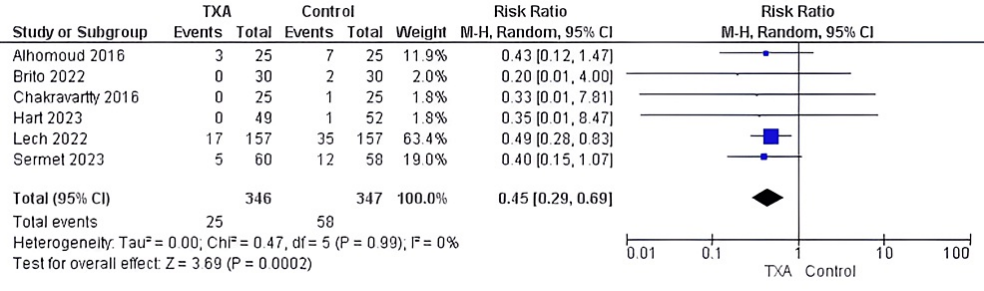
Forest plot of incidence bleeding events (postoperative) Included studies [18,20-22,28,29] TXA= tranexamic acid

Bleeding events: Analysis of six studies enrolling 693 patients [18,20-22,28,29] revealed that the TXA group was significantly associated with a 55% reduction in risk of postoperative bleeding events compared to the control group (RR= 0.45, 95%CI [0.29, 0.69], P=0.0002). The pooled studies were homogenous (I2 =0%) ( Figure 7).

**Figure 8:**
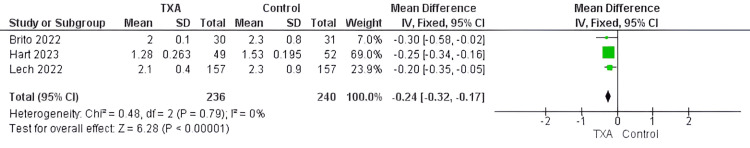
Forest plot of length of hospital stay (days) Included studies [22,28,29] TXA= tranexamic acid

Length of hospital stay (days): Analysis of three homogenous studies enrolling 476 patients [22,28,29] showed a significantly shorter hospital stay associated with the TXA group compared to the control group (MD=-0.24, 95%CI [-0.32, -0.17], P< 0.00001, I2 =0%) (Figure 8).

**Figure 9:**
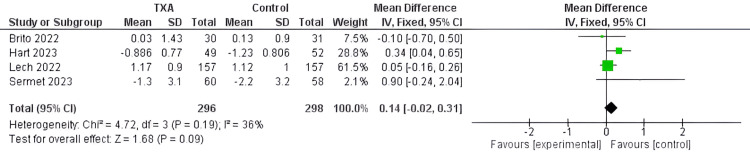
Forest plot of Change in hemoglobin (g per dl) Included studies [21,22,28,29] TXA= tranexamic acid

Change in hemoglobin (g per dl): There was no significant difference between the TXA and the control groups regarding pre-postoperative hemoglobin change according to the pooled 594 patients' data from four mildly heterogeneous studies [21,22,28,29] (MD=0.14, 95%CI[ -0.02, 0.31], P=0.09, and I2=36%) (Figure 9).

Discussion

Our study offers a fresh perspective on the most recent meta-analysis, incorporating two additional highly methodologically rigorous RCTs comprising data from 278 patients. This represents a population increase of over 58% compared to the previous study. We observed a significant advantage of the TXA group over the control group in terms of most intra-operative and postoperative outcomes. These include a reduced occurrence of intra-operative bleeding instances, a decrease in the volume of operative blood loss, a reduction in operative time, fewer postoperative bleeding events, and a shorter hospital stay. However, we found no significant difference between the TXA and control groups concerning the number of hemostasis clips used intraoperatively or the change in hemoglobin levels pre- and post-operation.

TXA was first invented by the husband-and-wife Okamoto and Okamoto in 1962 [30]. Since then, more research has been conducted regarding its action mechanistic and use cases, considering it one of the essential medicines and recommending it in various bleeding conditions [31-33]. The clotting and coagulation pathways aim to stop bleeding primarily through platelet activation, then secondarily through clotting factors activations, active thrombin formation, and ultimately, platelet plug formation. Fibrin augments the platelet plug by surrounding it in a network, an action that is physiologically counteracted by plasmin. TXA acts as a procoagulant through multiple mechanisms. It competitively inhibits the formation of the active form plasmin from plasminogen [34,35] and prevents interaction of plasmin with the formed fibrin network through synthesizing insoluble complex with the circulating plasmin [34]. Additionally, inhibition of plasmin formation limits the physiological inflammatory response, as plasmin is considered a proinflammatory protein that influences the generation of inflammatory cytokines, including interleukin 6 [36-38]. TXA could be administered orally, intramuscular (IM), intravenous (IV), through nebulization, and local application [39,40], in addition to IV push or intra-osseous in military situations [41]. It is considered a generally safe drug with mild adverse events, including vomiting, nausea, diarrhea, dizziness, hypotension, or visual problems [42]. Although very rare, some cases were reportedly associated with pulmonary embolism, vein thrombosis, or other thromboembolic adverse events [34]. Additionally, TXA was found to increase the rate of seizure attacks; the higher the dose, the higher the risk of developing seizures [43-45].

Mocanu and colleagues conducted a recent meta-analysis comprising data from 475 patients [19] who were enrolled in four moderate to low-quality studies [18,20,28,29] and underwent laparoscopic sleeve gastrectomy (LSG). They found TXA significantly associated with the reduction of postoperative bleeding by 60% compared to the placebo group (OR= 0.40; 95% CI [ 0.23-0.70]; P= 0.001). Our results aligned with them, finding a significant 55% reduction in the TXA group compared to the control group (RR= 0.45, 95%CI [0.29, 0.69], P=0.0002). Additionally, our meta-analysis found TXA significantly linked to a lower incidence of intraoperative bleeding, lower volume of blood loss during operation, and shorter operative duration.

Regarding patient demographics, our results were nearly consistent with what Mocanu reported [19]; the mean BMI spanned between 37.3 to 56.25 kg/m2, the mean age ranged between 33.5 and 43.25 years, and nearly 80% of the enrolled population was females. A literature review uncovered inconsistencies in the prevalence of obesity between males and females. Flegal and colleagues conducted a comprehensive survey spanning 10 years to investigate the prevalence of obesity among adults in the United States. Their findings indicated a slightly higher proportion of obesity in females than in males, approximately 40% compared to 35% [46]. Conversely, the proportion of obese individuals among Italian males exceeded that of females, with 51% compared to 34% [47].

Interestingly, no study has reported a four-fold higher prevalence of obesity in women compared to men, which is the same ratio at which obese patients undergo LSG surgery. Discrepancies between the actual sex-based prevalence of obesity and sex-based cases undergoing LSG surgery could be attributed to the fact that body image disturbances are more common in females, who are often dissatisfied with their weight and are more likely to try various diets to lose weight than males [48,49]. This was confirmed by Mousapour and team, who matched a cohort of 707 males to an equal number of females, considering various factors, including age and BMI. They followed them for 36 months after bariatric surgeries and found comparable safety and efficacy outcomes among males and females. They advised against considering patient sex when selecting cases for bariatric surgeries [50].

The potential for postoperative thromboembolic events at various time points following surgery remains a contentious issue. Over two decades ago, Sweetland and colleagues undertook a meta-analysis, incorporating studies published between 1996 and 2001, to evaluate the incidence of postoperative VTE events in middle-aged females. They included 947,454 females with an average age of 55.8 years and a BMI of 26 kg/m2. Among females who underwent day-case surgery compared to those who did not, the relative risks of VTE increased significantly, almost tenfold, for the first six weeks after surgery, about six times for 7-12 weeks post-surgery, 3.7 times for 4-6 months post-surgery, and 2.6 times for either 7-9 months or 10-12 months post-surgery [51]. Considering significant differences in surgical techniques, preoperative preparation, and postoperative care, as well as potential data omissions from retrospective studies, Singh et al. conducted a meta-analysis of prospective studies that included 20 or more venous thromboembolic (VTE) events following all types of surgeries, excluding obstetrics, neurology, pediatrics, and cardiac surgeries, published in or after the year 2000. They pooled postoperative data from 1,864,875 patients, tracked the incidence of VTE events, and identified 24,927 cases. All these cases occurred within the first month post-surgery, and no cases were identified after the 28th postoperative day. Approximately half of the cases occurred in the first week, with the remaining half occurring in the subsequent three weeks post-surgery [52,53].

Our meta-analysis found no instances of thromboembolic events during a follow-up period of at least three to six months. To our knowledge, no studies have demonstrated an increase in postoperative VTE rates following the implementation of TXA. Given these findings, considering our follow-up period and the absence of reported thromboembolic events, we argue that our study presents a compelling case for using TXA in LSG, with a negligible risk of thromboembolic events.

The main strengths of our study are that we executed the most comprehensive meta-analysis to date, examining the safety and efficacy of TXA in LSG, incorporating six studies that included a total of 753 LSG cases. Our study pooled data from two recently conducted high-quality RCTs, which resembled over 58% of our total population, strengthening the insights that could be derived from our findings. Five out of six included studies, which accounted for over 93% of the enrolled population, were deemed fair or high quality, according to the respected standardized quality appraisal tool. The eligible studies were carried out in different countries including Kuwait, Brazil, United Kingdom, Poland, Netherlands, and Türkiye, thereby enhancing the generalizability of our drawn results. Following the enrolled patients for a minimum of three to six months resulted in no reported case of VTE. This suggests a negligible risk for VTE among patients undergoing LSG and receiving TXA, considering the most recent meta-analysis evaluating VTE risk post-surgery. Based on the currently available evidence, TXA has a strong safety and efficacy profile for its use in patients undergoing LSG, with no reported severe adverse events and a significant reduction of bleeding events and volume of blood loss during and after LSG compared to the control group.

Yet, we encountered inevitable limitations. Alhomoud's study [20] exhibited a lack of methodological precision, as evidenced by the ambiguous risk of bias in most evaluated domains, potentially distorting the outcomes. The eligible studies were conducted in various countries, each with differing funding levels and quality of medical services. This led to inconsistencies in surgical procedures, definitions of bleeding, and reported blood loss volumes, which we could not account for, potentially impacting our findings. Moreover, the eligible studies did not uniformly provide data on other anticoagulant and antiplatelet medications, which could also influence our results.

## Conclusions

TXA demonstrates a robust safety and efficacy profile for its use in patients undergoing LSG, with no reported instances of thromboembolism and a significant reduction in surgical duration, hospitalization time, bleeding incidents, and volume of blood loss during and post-LSG compared to the control group. Variations in TXA administration regimens, bleeding definitions, procedural techniques, and potential confounders, including antiplatelet and anticoagulants, could not be accounted for, necessitating additional large-scale RCTs to address and bridge those knowledge gaps.
